# Psychological Profiling and Quantitative Sensory Testing Parameters as Predictors of Experimental Induced Muscle Pain

**DOI:** 10.1002/ejp.70363

**Published:** 2026-08-20

**Authors:** Sarah Guldberg, Mie Winther, Christine Rohde Jarding, Shukria Ismail Abukar Hassan, Emma Hertel, Kristian Kjær‐Staal Petersen

**Affiliations:** ^1^ Department of Health Science and Technology, Faculty of Medicine Aalborg University Gistrup Denmark; ^2^ Department of Material and Production, Center for Mathematical Modeling of Knee Osteoarthritis (MathKOA), Faculty of Engineering and Science Aalborg University Gistrup Denmark; ^3^ Department of Health Science and Technology, Center for Neuroplasticity and Pain (CNAP), SMI Aalborg University Gistrup Denmark

**Keywords:** delayed onset of muscle soreness, prediction of pain, psychological assessment, quantitative sensory testing

## Abstract

**Background:**

Pain is a multifactorial problem, and several pain mechanisms are likely contributing to why an individual might experience more pain than another person. The current study aimed to investigate whether pain sensitivity, assessed by quantitative sensory testing, and psychological factors could explain individual variability in pain intensity during experimentally induced Delayed Onset of Muscle Soreness (DOMS).

**Methods:**

Demographics, psychological distress, sleep quality, pain catastrophising, bodily sensation, alexithymia, pressure pain threshold, pain tolerance threshold, temporal summation of pain, and conditioned pain modulation were assessed at baseline in 32 healthy participants. Delayed onset of muscle soreness was induced using eccentric calf raises. During the following 3 days, the participants reported their pain intensity during muscle contraction (visual analogue scale, 0–10), sleep duration (hours), and sleep quality (visual analogue scale, 0–10).

**Results:**

Linear regression models with backwards elimination found that cuff pressure pain threshold, temporal summation of pain, and symptoms of anxiety and depression predicted 30.2% of the variation in peak pain intensity, while cuff pressure pain threshold and symptoms of anxiety and depression predicted 28.8% of the variation in cumulative pain intensity. Delayed onset of muscle soreness pain was not found to be mediated by sleep quality, sleep time or sleep efficiency.

**Conclusion:**

Psychological state and mechanisms indicative of pain sensitisation are related to the pain intensity during a bout of experimentally induced delayed onset of muscle soreness.

**Significance Statement:**

Pain is variable and an acute injury might be more painful to some than others. It is currently unclear what the underlying reasons for this variability are. This work demonstrates that increased sensitivity to pain and increase psychological load is associated with higher pain intensities following an experimental pain model. This suggests that certain people are more vulnerable to pain than others and that psychological and pain sensitivity measures might be measure to identify these pain vulnerable people.

## 1 Introduction

Chronic pain affects 20% of the global population (Goldberg and McGee 2011), with evidence suggesting demographic factors, such as gender, age and social class (Andersson et al. 1993), pain‐sensitising mechanisms (Graven‐Nielsen and Arendt‐Nielsen 2010), sleep (Chang et al. 2022) and psychological factors (Bushnell et al. 2013; Horsburgh et al. 2024) could be contributing elements to the transition from acute to chronic pain.

Assessments of the nervous system in terms of pain processes have been associated with chronic pain (Amiri et al. 2021; Suokas et al. 2012). Such assessments can be conducted using quantitative sensory testing (QST) (Mücke et al. 2021), with pressure‐based stimuli commonly used for evaluating deep‐tissue sensitivity and musculoskeletal pain (Jespersen et al. 2007; McPhee and Graven‐Nielsen 2019). Studies have identified measurements, such as pressure pain thresholds (PPTs), pain tolerance threshold (PTT), temporal summation of pain (TSP) and conditioned pain modulation (CPM), as potential outcome predictors of musculoskeletal pain across musculoskeletal conditions and clinical interventions, but with great variation (Georgopoulos et al. 2019; Petersen et al. 2021, 2023).

Epidemiological studies have found poor sleep quality to be associated with chronic pain development (McBeth et al. 2015). Likewise, sleep deprivation or fragmentation was found to reduce pain threshold and pain tolerance in experimental studies using healthy participants (Chang et al. 2022; Schrimpf et al. 2015), and potentially modulate central pain mechanisms (Hertel et al. 2023; Staffe et al. 2019). Furthermore, sleep deprivation has been associated with increased pain sensitivity during experimentally induced musculoskeletal injuries (Palsson et al. 2023).

In terms of psychological factors, pain catastrophising was found to be predictive of chronic pain and associated with increased pain intensity in chronic pain patients (Martinez‐Calderon et al. 2019). Additionally, higher scores of pain catastrophising and depression measurements have been associated with increased pain severity and long‐term pain persistence and predictive of musculoskeletal pain development within a 6‐month to 3‐year follow‐up (Edwards et al. 2011). Furthermore, elevated alexithymia has been linked to increased pain intensity in chronic pain patients compared to control groups (Aaron et al. 2019). Investigation of individual psychological predictors has resulted in variable association with chronic pain, while grouped psychological factors demonstrate medium‐to‐large effects (Jensen et al. 2011), supporting the use of a multimodal approach, including variables such as psychological factors and QST (Giordano et al. 2024; Hertel et al. 2024).

Delayed onset muscle soreness (DOMS) is an experimental pain model mimicking an acute injury (Bishop et al. 2011). Previous studies have shown that psychological factors alone (Niederstrasser et al. 2014, 2015) or combined with QST measurements (Kristensen et al. 2021; McPhee and Graven‐Nielsen 2019) are predictive of DOMS pain intensity, but predictive values and relative importance of individual factors vary between individual studies. This highlights the need for further investigation into identified and new possible variables to establish a more definitive and stronger predictive model. As such, the current study aimed to investigate whether QST measurements combined with a broad range of psychological factors could predict DOMS pain intensity.

## 2 Methods

Participants were assessed during one experimental session in which they filled in questionnaires regarding baseline information, psychological symptoms, sleep, bodily sensations, and alexithymia, respectively. Following this, the participants were assessed using computer‐controlled cuff‐pressure algometry to determine their PPT, PTT, TSP and CPM. Participants then performed eccentric calf raises on their dominant legs to evoke DOMS.

**FIGURE 1:**
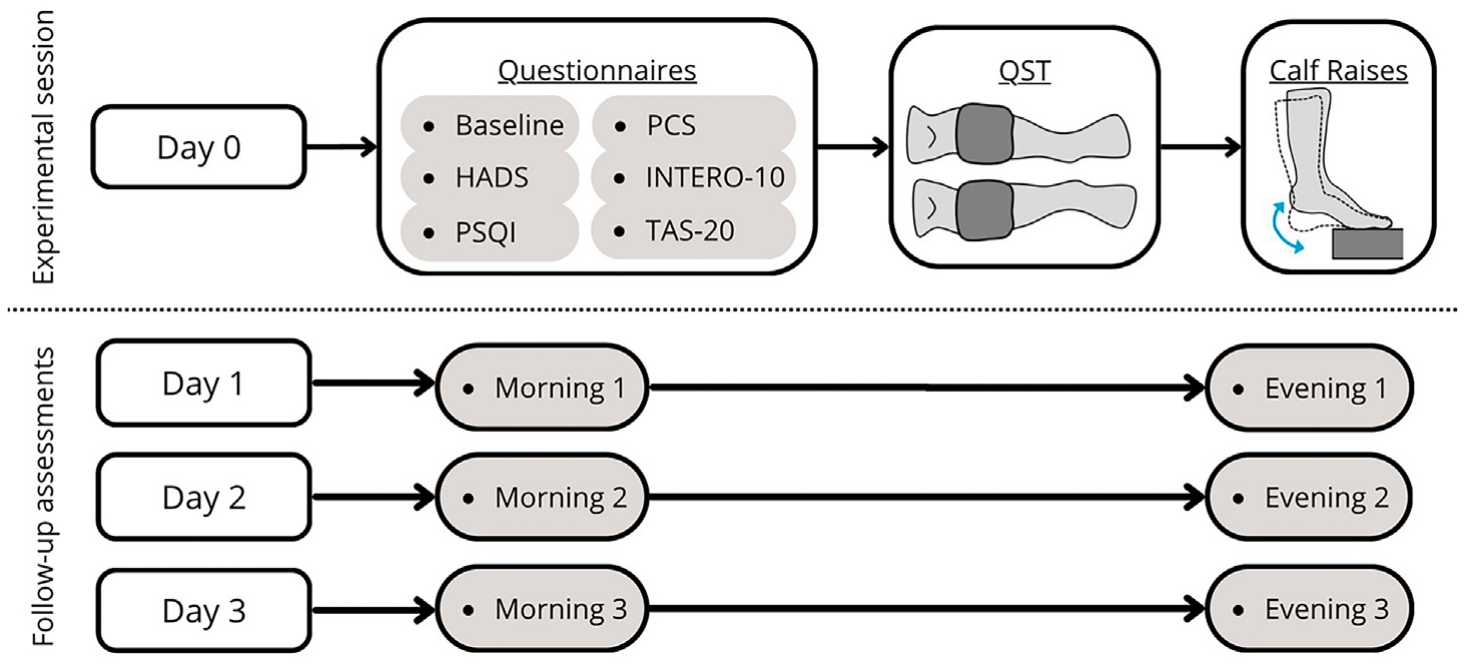
Flowchart of the experimental setup for the participants for the experimental session and the three follow‐up days. The experimental session includes questionnaires: Baseline, baseline information; HADS, Hospital Anxiety and Depression Scale; INTERO‐10, Embodied Interoception Questionnaire; PCS, Pain Catastrophizing Scale; PSQI, Pittsburgh Sleep Quality Index; QST, quantitative sensory testing and calf raises; TAS‐20, Toronto Alexithymia Scale 20. Days 1, 2, and 3 include answering the follow‐up assessment in the mornings (Morning 1–3) and evenings (Evening 1–3).

During the following 3 days, the participants reported a total of six follow‐up assessments of their pain level in their dominant calf when flexing and information regarding their sleep. The follow‐up assessments were scheduled in the morning and evenings of the 3 days (Figure 1). The study was approved by The North Danish Regional Committee on Health Research Ethics (N‐20240036) and was conducted at Aalborg University, Aalborg, Denmark. The recruitment was initiated on November 1 and was completed on November 29, 2024. The data presented here are novel and have not been published previously.

### 2.1 Participants

Healthy participants within the age range of 18–30 years were recruited from Aalborg University, Aalborg, Denmark and the wider community. Individuals were excluded if they reported previous or current chronic neurological or musculoskeletal conditions; psychiatric diagnoses; consumption of painkillers on the day of the experiment; current use of pharmaceuticals which could influence the trial; current or recent history of acute or chronic pain. Participants who reported a pain level at the calf of ≥ 2 on a visual analogue scale (VAS, 0 cm: no pain; 10 cm: worst pain imaginable) at the time of recruitment or failed to complete all follow‐up questionnaires were also excluded. Eligible participants received verbal and written information and provided written informed consent before participating.

### 2.2 Questionnaires

The participants completed a demographic questionnaire regarding age, weight, height, and leg dominance. Additionally, they completed a baseline information questionnaire in which they reported their current pain level in their dominant leg when flexing using a visual analogue scale (VAS, 0 cm: no pain, 10 cm: worst pain imaginable), the time they went to bed, began trying to fall asleep, and woke up as well as rated their sleep quality (VAS, 0 cm: worst quality of sleep imaginable, 10 cm: best quality of sleep imaginable). Moreover, the participants completed the following validated questionnaires: The Hospital Anxiety and Depression Scale (HADS) to measure symptoms of anxiety and depression in two subscales each with scores between 0 and 21 where higher scores indicate more severe symptoms (Bjelland et al. 2002; Christensen et al. 2020); The Pittsburgh Sleep Quality Index (PSQI) to measure sleep quality on a scale from 0 to 21 with higher scores indicating worse sleep quality (Buysse et al. 1989); The Pain Catastrophizing Scale (PCS) to measure pain‐related thoughts and distress on a scale from 0 to 51 with higher scores indicating a higher degree of pain catastrophising (Sullivan et al. 1995); The Embodied Interoception Questionnaire (INTERO‐10) to measure awareness of internal physiological sensations including 10 items rated on a VAS with higher ratings denoting greater interoceptive awareness (Musculus et al. 2021); The Toronto Alexithymia Scale 20 (TAS‐20) to measure the presence and severity of alexithymia on a scale from 20 to 100, with higher scores indicating greater severity of alexithymia (Bressi et al. 1996).

### 2.3 Computer‐Controlled Cuff Pressure Algometry

A computer‐controlled cuff pressure algometer (Cortex Technology, Hobro, Denmark and Aalborg University, Aalborg, Denmark) and an electronic VAS (Aalborg University, Aalborg, Denmark) were utilised to assess cuff PPT (cPPT), cuff PTT (cPTT), TSP and CPM. The cuffs (VBM, Sulz, a. N., Germany) were placed on the widest part of the participants' calves following previously reported protocols (Kristensen et al. 2021; Petersen, Olesen, et al. 2019; Petersen, Simonsen, et al. 2019).

#### 2.3.1 Cuff Pressure Pain and Tolerance Threshold

A ramped test with a constant cuff inflation rate of 1 kPa/s and a safety limit of 100 kPa was conducted to assess cPPT and cPTT in each leg separately, starting with the dominant leg. The participants were instructed to rate the pain produced by the pressure using the VAS (0 cm: no pain, 10 cm: worst pain imaginable) once the pressure became painful, and to continue the rating throughout the procedure. If the pressure became intolerable, they pressed a stop button. The cPPT was defined as the pressure (kPa) at which the participant reached 1 cm on the VAS, while the cPTT was defined as the maximum pressure (kPa) the participant could tolerate, following previous reports (Kristensen et al. 2021; Petersen, Simonsen, et al. 2019).

#### 2.3.2 Temporal Summation of Pain

Ten consecutive inflations at cPTT intensity were applied to the dominant leg (interval: 1 s on, 1 s off), creating a pulsating sensation. Participants rated the pain of the first inflation and adjusted their rating for each of the following nine inflations. TSP was calculated by comparing the average VAS scores of the initial four stimuli with the average score of the last three stimuli (Graven‐Nielsen et al. 2015; Kristensen et al. 2021; Petersen, Simonsen, et al. 2019).

#### 2.3.3 Conditioned Pain Modulation

The cuff on the non‐dominant leg was inflated to a steady conditioning pressure at 70% of the cPPT level. Simultaneously, the cuff on the dominant leg was inflated continuously at 1 kPa/s. Participants rated pain in the dominant leg using the VAS while attempting to disregard pain in the non‐dominant leg. CPM was calculated as the difference in cPPT measured with the conditioning stimulus and cPPT without the conditioning stimulus (Petersen et al. 2016, 2025).

### 2.4 Experimental Muscle Pain

Unilateral calf raises were performed on the dominant leg without shoes to induce DOMS. Participants positioned the ball of their foot on a step, allowing the heel to hang off the edge. Without offloading weight from the working leg, participants were allowed to use a railing for balance. Participants were instructed to raise and lower their heels through the full range of motion (Kristensen et al. 2021). The exercise was completed in four sets, each with up to 30 repetitions and with verbal encouragement from the investigator. Additionally, each set could be terminated by the participants if they reached fatigue or by the investigator if the participant could no longer reach the full range of motion in two consecutive calf raises. The maximum rest period between two sets was 2 min.

### 2.5 Follow‐Up Assessments

The participants completed six follow‐up assessments over the following 3 days in the mornings (M1‐3) and evenings (E1‐3). For each assessment, the participants reported their current pain level in their dominant leg when flexing (VAS, 0 cm: no pain, 10 cm: worst pain imaginable), the time they went to bed, began trying to fall asleep, and woke up as well as their sleep quality (VAS, 0 cm: worst quality of sleep imaginable, 10 cm: best quality of sleep imaginable). The participants were asked not to exercise between the onset of DOMS and until the completion of the study.

### 2.6 Statistical Analysis

An a priori power calculation was performed based on previous experimental DOMS studies using similar protocols and outcome measures (Kristensen et al. 2021; McPhee and Graven‐Nielsen 2019). With an effect size of 0.35, an alpha level of 0.05, a power of 0.8, 9 parameters in the initial model and an expected 1 independent parameter following backwards elimination, a total of 25 participants were required. To account for dropouts and exclusion, 42 participants were recruited.

Unless otherwise stated, the data are presented as mean ± standard deviation (SD). Repeated measures (RM) ANOVA was used to investigate changes in DOMS VAS scores and sleep quality from Days 0 to 3. Bonferroni corrected post hoc tests were conducted if significant main effects were detected. Linear regression models were used to explain variability in peak DOMS VAS scores and cumulative DOMS VAS scores. Both models included independent baseline variables: cPPT, cPTT, TSP, CPM, HADS, PSQI, PCS, INTERO‐10, and TAS‐20. There were no indications of collinearity or multicollinearity between any of the independent variables, as all collinearity levels were above 0.1 and the variability inflation factor (VIF) levels were below 10. Backward selection was used to identify the best model from the independent variables. The number of iterations was reported. The independent variables identified as important in both regression models were then used as covariates in the RM‐ANOVA described above. The PROCESS Macro for SPSS was used to investigate the potential of sleep parameters as mediators of DOMS development during the follow‐up days. Three different sleep parameters were used as mediators in individual models and for each of the three nights during follow‐up: (1) sleep quality, (2) sleep time, and (3) sleep efficiency during Nights 1–3. The statistical analyses were performed using SPSS (IBM SPSS Statistics for Windows, version 28.0).

## 3 Results

### 3.1 Baseline Characteristics

Forty‐two participants were recruited. Ten participants were excluded as they reported pain in the dominant calf (VAS ≤ 2, *n* = 7) at baseline or failed to complete all six follow‐up questionnaires (*n* = 3). The baseline characteristics of the included thirty‐two participants are presented in Table 1.

**TABLE 1:** Demographic and baseline data for all thirty‐two participants included for analysis.

Baseline characteristic	
Age (years)	24.1 ± 2.7
BMI (kg/m^2^)	23.4 ± 3.6
HADS	8.5 ± 5.1
PSQI	5.8 ± 2.9
PCS	8.3 ± 7.5
INTERO‐10	30.3 ± 12.9
TAS‐20	55.5 ± 8.7
cPPT (kPa)	32.9 ± 13.8
cPTT (kPa)	78.7 ± 21.1
TSP (ΔVAS)	1.2 ± 0.8
CPM (ΔkPa)	12.9 ± 11.7

Abbreviations: BMI, body mass index; cPPT, cuff pressure pain threshold; cPTT, cuff pain tolerance threshold; CPM, conditioned pain modulation; HADS, The Hospital Anxiety and Depression Scale; INTERO‐10, The Embodied Interoception Questionnaire; PCS, The Pain Catastrophizing Scale; PSQI, Pittsburgh Sleep Quality Index; TAS‐20, The Toronto Alexithymia Scale 20; TSP, temporal summation of pain.

### 3.2 Characteristics of DOMS Development

**FIGURE 2:**
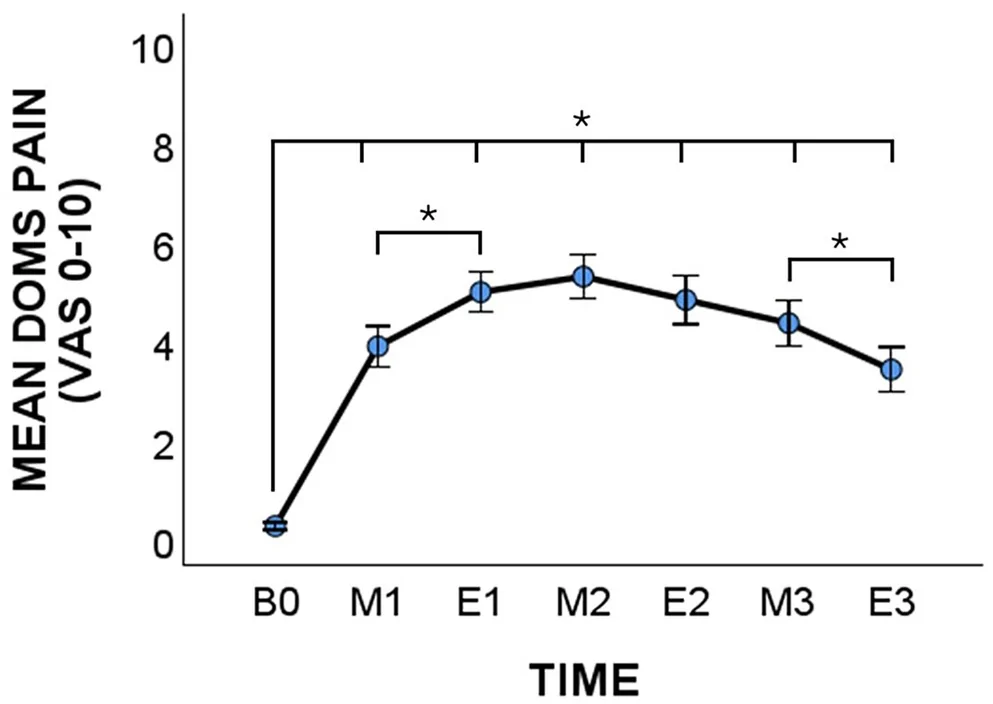
Graph of the mean pain level (visual analogue scale 0–10; 0 ‘no pain’, 10 ‘worst pain’) in the participants' dominant leg when flexing at baseline (B0) and post DOMS, delayed onset muscle soreness measured during three follow‐up days at the morning (M1–M3; Mornings 1–3) and evening (E1‐E3; Evenings 1–3).

The mean number of repetitions in each of the four sets of exercise, starting from the first set, was 26.60 (5.58), 23.79 (7.18), 22.79 (7.51), and 22.19 (8.41). The mean peak pain was 5.86 (2.36), and most participants reported peak pain in Evening 2 (*n* = 20). The cumulative pain was 22.43 (10.52). RM‐ANOVA found that DOMS VAS ratings changed significantly over time (F_3.35,103.95_ = 39.70, *p* < 0.001). Post hoc tests (Bonferroni corrected) revealed a significant difference in DOMS VAS ratings from baseline to all other time points (*p* < 0.001) as follows: as seen in Figure 2. Further, DOMS VAS ratings significantly increased from Morning 1 (M1) to Evening 1 (E1) (*p* < 0.007). Finally, pain on Evening 3 (E3) was significantly lower than Morning 3 (M3) (*p* < 0.01) but still significantly increased compared to baseline (*p* < 0.001).

### 3.3 Effects on Sleep Quality on DOMS

The development of DOMS pain from day to day was not mediated by sleep quality, sleep time or sleep efficiency. RM‐ANOVA test found no significant effect of sleep quality on DOMS VAS ratings (*p* > 0.05). Additionally, the direct effect of both sleep time and sleep efficacy on the next morning's DOMS VAS ratings was investigated across all three consecutive days; however, no significant relations were found (*p* > 0.05). These were changes in the quality of sleep over time.

### 3.4 Predicting DOMS Intensity Using Baseline Characteristics

#### 3.4.1 Peak DOMS Pain

The initial model included cPPT, cPTT, TSP, CPM, HADS, PSQI, PCS, INTERO‐10 and TAS‐20 and explained 45.2% of the variability in peak DOMS pain (F_9,22_ = 2.02, *p* = 0.087) with an adjusted *R* ^2^ of 22.8%. After seven iterations, the final model included cPPT, TSP, and HADS and significantly explained 36.9% of the variability in peak DOMS pain (F_3,28_ = 5.47, *p* < 0.01) with an adjusted *R* ^2^ of 30.2%. The initial and final models are shown in Table 2.

**TABLE 2:** Initial and final multiple linear regression model with backwards elimination for peak DOMS, delayed onset muscle soreness pain. The variables included were the questionnaire data and quantitative sensory testing parameters.

Model	Independent variable	β	*p*	Adjusted *R* ^2^
**Initial**				0.228
	**Constant**		0.02	
	cPPT	−0.54	0.04	
	cPTT	0.01	0.98	
	TSP	0.32	0.08	
	CPM	−0.06	0.78	
	PSQI	0.19	0.34	
	PCS	−0.13	0.48	
	INTERO‐10	0.28	0.18	
	TAS‐20	−0.03	0.89	
	HADS	−0.47	0.02	
**Final** *7th iteration*				0.302
	**Constant**		< 0.001	
	cPPT	−0.44	0.007	
	TSP	0.22	0.155	
	HADS	−0.37	0.020	

Abbreviations: CPM, conditioned pain modulation; cPPT, cuff pressure pain threshold; cPPT, cuff pressure tolerance threshold; HADS, The Hospital Anxiety and Depression Scale; INTERO‐10, The Embodied Interoception Questionnaire; PCS, The Pain Catastrophizing Scale; PSQI, Pittsburgh Sleep Quality Index; TAS‐20, The Toronto Alexithymia Scale 20; TSP, temporal summation of pain.

#### 3.4.2 Cumulative DOMS Pain

The initial model included cPPT, cPTT, TSP, CPM, HADS, PSQI, PCS, INTERO‐10 and TAS‐20 and explained 48.0% of the variability in cumulative DOMS pain (F_9,22_ = 2.26, *p* = 0.058) with an adjusted *R* ^2^ of 26.7%. After eight iterations, the final model included cPPT and HADS and explained 33.4% of the variability in cumulative DOMS pain (F_2,29_ = 7.28, *p* < 0.01) with an adjusted *R* ^2^ of 28.8%. The initial and final models are shown in Table 3.

**TABLE 3:** The initial and final multiple linear regression model with backwards elimination for cumulative DOMS, delayed onset muscle soreness pain. The variables included were the questionnaire data and quantitative sensory testing parameters.

Model	Independent variable	β	*p*	Adjusted *R* ^2^
**Initial**				0.267
	**Constant**		0.02	
	cPPT	−0.57	0.03	
	cPTT	0.05	0.82	
	TSP	0.29	0.10	
	CPM	−0.02	0.91	
	PSQI	0.23	0.23	
	PCS	−0.14	0.43	
	INTERO‐10	0.33	0.10	
	TAS‐20	−0.09	0.63	
	HADS	−0.49	0.02	
**Final** *8th iteration*				0.288
	**Constant**		< 0.001	
	cPPT	−0.43	0.008	
	HADS	−0.40	0.013	

Abbreviations: CPM, conditioned pain modulation; cPPT, cuff pressure pain threshold; cPPT, cuff pressure tolerance threshold; HADS, The Hospital Anxiety and Depression Scale; INTERO‐10, The Embodied Interoception Questionnaire; PCS, The Pain Catastrophizing Scale; PSQI, Pittsburgh Sleep Quality Index; TAS‐20, The Toronto Alexithymia Scale 20; TSP, temporal summation of pain.

### 3.5 DOMS Development Adjusted for Baseline Parameters

An explorative analysis was conducted, adjusting the RM‐ANOVA models to investigate changes in DOMS over time for the variables that emerged as significant in the regression models. When adjusting for baseline cPPT as a covariate in the RM‐ANOVA, the effect of time on DOMS VAS ratings is not significant (F_3.21,96.31_ = 1.64, *p* = 0.139), suggesting baseline cPPT accounts for much of the variance in DOMS VAS ratings over time. When adjusting for baseline HADS as a covariate in the RM‐ANOVA, the effect of time on DOMS VAS remains significant (F_12.19,104.20_ = 3.19, *p* < 0.05). This suggests the baseline HADS may not affect the DOMS VAS rating over time.

## 4 Discussion

The current exploratory study found that a combination of measures of pain sensitivity and symptoms of anxiety and depression predicted DOMS pain intensity in healthy subjects. This supports the notion that the experience of pain is a multimodal phenomenon (Kristensen et al. 2021; McPhee and Graven‐Nielsen 2019). In the current study, cPPT emerged as the strongest predictor for DOMS. In addition, the current study was unable to demonstrate that daily variation in sleep quality mediated DOMS pain intensities.

### 4.1 Characteristics of DOMS Pain

The current study used calf raises as the experimental pain model to induce DOMS. The current study found that the pain seems to increase progressively during the first 24 h (M1‐E1), followed by a short stable period (M2–M3), and then decrease at the final follow‐up at the 72‐h mark (E3). This indicates that DOMS peaks between 24 h (E1) and 48 h (E2) after the exercise performance, and that, on average, the DOMS pain requires more than 72 h to dissipate completely. These results extend the findings of previous literature by showing the temporal development of DOMS in which the peak pain varies on an individual basis, emphasising the importance of including more post‐induction assessments.

### 4.2 Baseline Predictors of DOMS Pain

The current study identified cPPT as the strongest predictor of DOMS pain, with a lower cPPT associated with increased DOMS pain intensity. cPPT is considered a measure of localised hyperalgesia at the calf (Graven‐Nielsen and Arendt‐Nielsen 2010) and therefore, this finding suggests that higher localised hyperalgesia in a muscle could be a risk factor for more intense pain when the muscle is injured. Previous studies have found lower pain thresholds in patients with chronic musculoskeletal pain compared to healthy controls (Arendt‐Nielsen et al. 2010; Jespersen et al. 2007; Petzke et al. 2003). Furthermore, a lower baseline pain threshold in patients with musculoskeletal conditions was found to be predictive of worse outcomes following clinical interventions, such as higher pain intensity and disability (Georgopoulos et al. 2019; Sterling et al. 2011) as well as increased risks of developing post‐operative chronic pain (Petersen et al. 2021). Similarly, experimental multivariable studies using DOMS have also identified low PPT associated with higher DOMS pain (Hertel et al. 2025; Kodesh et al. 2022), however, other studies have not been able to identify a significant association (Kristensen et al. 2021; McPhee and Graven‐Nielsen 2019). This could imply that the predictive role of PPT may depend on a specific combination of variables included in the model, emphasising the need for future investigations to explore the role of PPT in multimodal predictive models. TSP also contributed to explaining the variability in peak DOMS pain, which is in accordance with previous literature, finding facilitated TSP associated with increased clinical (Hertel et al. 2024) and experimental pain (Kristensen et al. 2021; McPhee and Graven‐Nielsen 2019). This indicates that individual variations in pain sensitivity likely encode for vulnerability to acute pain following eccentric exercise, and indications of increased pain sensitivity seem to characterise an individual more likely to develop higher DOMS pain.

HADS was identified as a significant predictor of DOMS, with higher levels of anxiety and depression associated with lower DOMS intensity. This appears counterintuitive, however, it may reflect a complex interplay between psychological states and acute pain perception in otherwise healthy volunteers. Similarly, previous experimental DOMS studies found positive mood states predictive of increased severity of DOMS pain (Hertel et al. 2025; Kristensen et al. 2021). Contrarily, Fabero‐Garrido et al. 2022 reported a significant relationship between greater distress (HADS) and higher DOMS intensity in the cervical region in healthy individuals (Fabero‐Garrido et al. 2022). On the other hand, George et al. (2007) found no association between anxiety (State–Trait Anxiety Questionnaire) and pain intensity following DOMS induction in the external rotator shoulder muscles (George et al. 2007). These discrepancies underscore the need for further research to clarify how emotional states influence acute pain perception.

The combination of cPPT, TSP and HADS was predictive of DOMS pain, supporting that a multimodal approach to explain variability in pain is beneficial, which is consistent with findings from previous research (Giordano et al. 2024; Hertel et al. 2024; Kristensen et al. 2021; Petersen et al. 2026). Previous research has found pain catastrophising to be predictive of increased pain intensity and disability in chronic pain patients; however, the strength of the conclusion is limited by the quality of the studies (Martinez‐Calderon et al. 2019). Additionally, pain catastrophising demonstrated varying degrees of explanatory value within multimodal predictive models in a clinical osteoarthritis cohort (Hertel et al. 2024). The current study did not find a significant relationship between PCS and DOMS, and previous research on the effects of pain catastrophising on experimental pain in healthy volunteers is also inconclusive, ranging from no effect (Kristensen et al. 2021; McPhee and Graven‐Nielsen 2019) to a strong effect (Niederstrasser et al. 2014, 2015). This could suggest other factors may be influencing the degree to which pain catastrophising affects DOMS pain.

### 4.3 Sleep Quality and DOMS Pain

Previous poor sleep quality has been linked to increased pain sensitivity in chronic pain conditions (Koffel et al. 2016; Wiklund et al. 2020). Similarly, findings from previous research show that experimental sleep disruptions are associated with increased pain sensitivity in healthy volunteers (Hertel et al. 2024; Palsson et al. 2023; Schuh‐Hofer et al. 2013). Nevertheless, the current study found no significant associations between DOMS and the included sleep metrics. DOMS generally evoke movement‐evoked acute pain (Kodesh et al. 2022; Matsuda et al. 2015), which is likely insufficient in both intensity and duration to provoke any noticeable changes in sleep quality. This was also the case in the current study, finding no significant differences in sleep quality before and after induction of DOMS. To investigate the effects of sleep on experimental pain development, future studies should employ models with pain sufficient to disturb sleep or combine the DOMS models with experimental sleep disturbance, which has previously been shown to increase DOMS responses (Palsson et al. 2023).

### 4.4 Multimodal Prediction of DOMS Pain

It could be speculated that the interplay between the predictors influences the total prediction more than any single variable alone, and that some variables depend on co‐occurrence to predict pain levels. For instance, Niederstrasser et al. (2015) found pain catastrophising as a significant predictor in the presence of high anxiety, potentially indicating an interactive effect (Niederstrasser et al. 2015). This could offer a potential explanation for inconsistent findings across studies. Overall, the findings of the current study emphasise the importance of including multiple variables across multiple domains in predictive models. Future research should focus on incorporating sensory testing, psychological factors, and biological biomarkers, such as inflammation and epigenetics, into pain investigation.

### 4.5 Limitations

The linear regression model in the current study utilised backward elimination to gauge the most important variables. This method does not allow re‐entering variables and might miss variables that would be significant in later iterations. Nevertheless, this method has frequently been used in similar studies (Kristensen et al. 2021). Future research could investigate alternative or supplementary methods to account for variable interactions or permit excluded predictors to be re‐entered, enhancing the model's accuracy and reliability.

## 5 Conclusion

The current study demonstrated that cuff pressure pain threshold, temporal summation of pain and symptoms of anxiety and depression explained 30.2% of the variability in peak muscle pain intensity after eccentric calf raises, while a cuff pressure pain threshold and symptoms of anxiety and depression explained 28.8% of the variability in the cumulative muscle pain intensity. Lower pressure pain threshold facilitated temporal summation of pain, and decreased anxiety and depression scores were linked to higher DOMS pain intensities. These findings highlight the need for a multimodal approach in predicting pain.

## Author Contributions

This study was designed by K.K.‐S.P., and E.H. The experiments were performed by S.G., M.W., C.R.J., and S.I.A.H. The data were analysed by S.G., M.W., C.R.J., and S.I.A.H., and the results were critically examined by all authors. S.G. had a primary role in preparing the manuscript, which was edited by E.H. and K.K.‐S.P. All authors have approved the final version of the manuscript and agree to be accountable for all aspects of the work.

## Funding

Center for Neuroplasticity and Pain (CNAP) is supported by the Danish National Research Foundation (DNRF121). The Center for Mathematical Modelling of Knee Osteoarthritis (MathKOA) is funded by the Novo Nordisk Foundation (NNF21OC0065373).

## Disclosure

No AI was used for this manuscript.

## Conflicts of Interest

The authors declare no conflicts of interest.

## Supporting information


**Data S1:** STROBE statement—checklist of items that should be included in reports of *cohort studies*.

## Data Availability

Data supporting the findings of the current work are available from the corresponding author upon reasonable request.
